# Blue Light Defocus Induces A Positive Effect on Refractive Status and Ocular Health: A Randomized Crossover Trial

**DOI:** 10.1002/gch2.202500222

**Published:** 2025-11-14

**Authors:** Jinfang Wu, Ze‐Hua Xu, Yuanyuan Miao, Xinyao Zheng, Lan Yang, Cong Wang, Jason C. Yam, Zi‐Bing Jin

**Affiliations:** ^1^ Beijing Institute of Ophthalmology Beijing Tongren Eye Center Beijing Ophthalmology & Visual Sciences Key Laboratory Beijing Tongren Hospital Capital Medical University Beijing China; ^2^ Eye Hospital and School of Ophthalmology and Optometry Wenzhou Medical University Wenzhou Zhejiang China; ^3^ Department of Ophthalmology and Visual Sciences The Chinese University of Hong Kong Hong Kong China; ^4^ Hong Kong Eye Hospital Hong Kong China; ^5^ Department of Ophthalmology and Visual Sciences Prince of Wales Hospital Hong Kong China; ^6^ Department of Ophthalmology Hong Kong Children's Hospital Hong Kong China; ^7^ Hong Kong Hub of Paediatric Excellence The Chinese University of Hong Kong Hong Kong China; ^8^ Joint Shantou International Eye Center of Shantou University and the Chinese University of Hong Kong Shantou China

**Keywords:** blue light, defocus, display technology, myopia

## Abstract

This randomized crossover trial investigates the effects of blue light defocus display technology on refractive status, axial length (AL), retinal blood flow, and visual function in adults. Twenty‐one participants completed all four interventions: 0D, 1D, 2D defocus, and 1D defocus with 30 % blue light filtering (1D+BLF) in a randomized order during standardized visual tasks. Pre‐ and post‐task assessments include refraction, AL, choroidal thickness (ChT), retinal defocus, reading efficiency, and visual fatigue. Results demonstrate that 1D defocus reduces spherical equivalent refraction (SER) (−4.35 ± 2.66 D to −4.21 ± 2.66 D, *P* = 0.045) and increases ChT (*P* = 0.003), while 1D+BLF induces axial elongation (*P* = 0.026). Both 1D and 2D defocus are linked to increased ChT, whereas 0D and 1D+BLF groups exhibited hyperopic defocus trends. Reading speed and efficiency improve in the 1D group (*p* < 0.05), while visual fatigue and blink frequency increase significantly in the 0D group (*p* = 0.001). Linear regression identifies correlations between defocus and changes in choroidal volume, near convergence, and fusional reserves. These findings suggest blue light defocus technology may help mitigate hyperopic defocus, influence retinal perfusion, and alleviate visual fatigue, supporting its potential role in myopia prevention. Further validation in diverse populations and long‐term studies is warranted.

## Graphical abstract

This graphical abstract illustrates that varying degrees of intensity of defocus are associated with choroidal thickness, myopic defocus shift, and manifestations of visual fatigue, along with alterations in accommodation and convergence functions. Preliminary evidence suggests that blue light defocus technology could serve as a potential intervention for ocular health optimization, though further clinical validation is warranted.

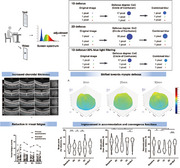

## 1 Introduction

Myopia has emerged as a significant public health challenge in the 21st century, with its prevalence escalating dramatically over recent decades. The progression of myopia is characterized by ocular axial elongation, leading to a decline in distance vision and structural changes, including thinning of the choroid and sclera [1, 2, 3]. These alterations significantly increase the risk of retinal degeneration and detachment [4, 5].

A fundamental question in understanding myopia is the mechanisms that drive the eye away from its normative developmental trajectory. This deviation is often attributed to sustained near work, particularly in modern lifestyles that require prolonged visual engagement at close distances. The alarming rise in myopia prevalence correlates with modern lifestyle shifts, including increased near work (e.g., screen use) and reduced outdoor activity, alongside genetic predisposition. The emmetropization process relies heavily on visual cues for focus adjustment [6]. However, contemporary environments present complex optical conditions that disrupt this delicate process. Various display devices—including smartphones, computers, televisions, and tablets—have become integral to daily life, with a substantial proportion of daily activities conducted through these screens. Extensive research has explored the light effects on ocular development, particularly the visual cues that guide changes in axial length (AL) [7, 8]. Factors, such as defocus, spherical aberration, coma, trefoil aberration, longitudinal chromatic aberration (LCA), contrast sensitivity, and spatial resolution [9] significantly influence the onset and progression of myopia [9, 10].

Among these factors, LCA has been extensively investigated as a critical visual cue [8, 11, 12, 13]. Nearly all vertebrate eyes exhibit pronounced LCA effects, typically demonstrating approximately two to three diopters of LCA within the visible spectrum [8]. Longer wavelengths of light (red) tend to focus further in the eye, while shorter wavelengths (blue) focus closer [14]. Many studies have manipulated focal planes using single wavelengths to induce changes in AL, while others have enhanced specific wavelengths to facilitate adaptive changes in the eye [15]. However, results have been inconsistent. For instance, Verkicharla et al. found that exposure to red and green light resulted in axial elongation in young individuals, while blue light appeared to inhibit this elongation [10, 12]. Similarly, Wang et al. reported that blue light exposure improved choroidal thickness (ChT) and blood perfusion, suppressing myopia progression. In contrast, other studies indicated that narrowband red light exposure could lead to hyperopia, while narrowband blue light fails to maintain emmetropia [16, 17]. The role of LCA in modulating axial changes remains debated, with some studies proposing it as a primary driver of emmetropization, while others suggest it acts as a secondary cue [18]. Nonetheless, it is unequivocal that the LCA mechanism is vital for controlling refractive statuses and axial changes [13], particularly in a modern society inundated with screen displays [19].

Considering this research background, the present study constructs models based on varying degrees of blue light defocus and intensity, simulating +1D and +2D blue defocus conditions, along with a comprehensive model combining +1D blue defocus with 30 % blue light filtering. These models were integrated with systematic clinical trials to investigate how different defocus levels and intensities of blue light affect changes in AL. Our research aims to provide insights into the influence of spectral stimulation on ocular growth and may establish effective strategies to regulate or mitigate myopia progression.

## 2 Results

**Figure 1:**
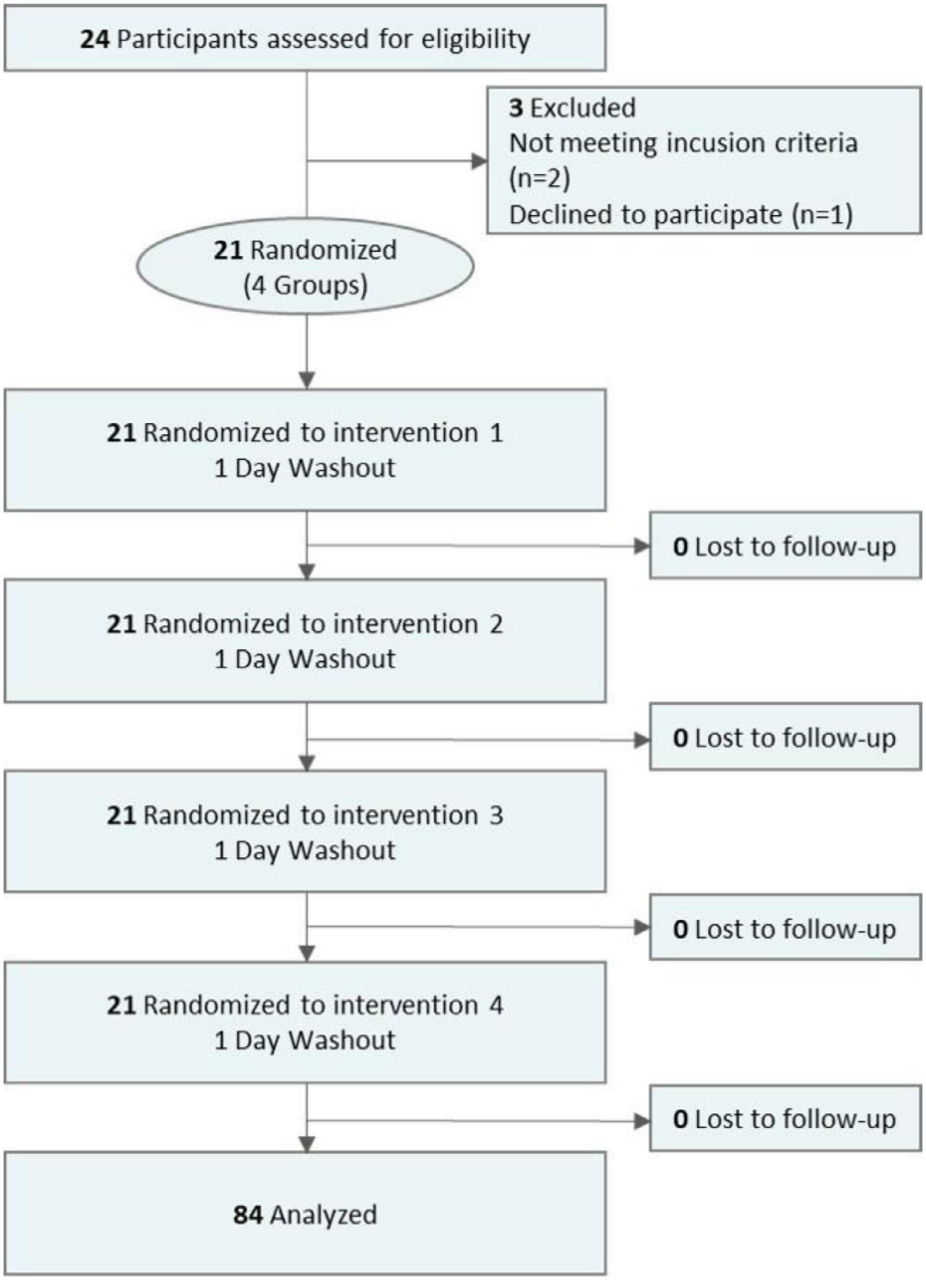
CONSORT overview of study participation.

Participant recruitment for the study occurred between 01‐06‐2024 and 30‐08‐2024, with 24 participants enrolled. Twenty‐one participants successfully completed the full study protocol (Figure 1). The baseline demographic and clinical characteristics of the study participants are comprehensively presented in Table 1. This study enrolled participants with low, moderate, and high myopia at proportions of 42.86 %, 38.09 %, and 19.05 %, respectively, mirroring the national prevalence among adults. The mean refractive error for the right was −4.24 ± 2.62 D, and for the left, −3.76 ± 2.66 D. The mean ChT at the fovea was 303.24 ± 100.11 µm for the right and 307.05 ± 78.03 µm for the left.

**Table 1:** Participant item distributions.

Participant Item		Distribution
Anisometropia		<1.5D
Intraocular Pressure		14–20mmHg
Diopter		−3.00D < SE ≤ −0.50D: 42.86% −6.00D < SE ≤ −3.00D: 38.09% ≤ −6.00D: 19.05%
Corrected Visual Acuity		≤ 0.0 logMAR
Male‐female Ratio		Males: 9 Females: 12
Dominant Eye		Right eye: 15 Left eye: 6

### 2.1 Changes in ChT and Volume

**Figure 2:**
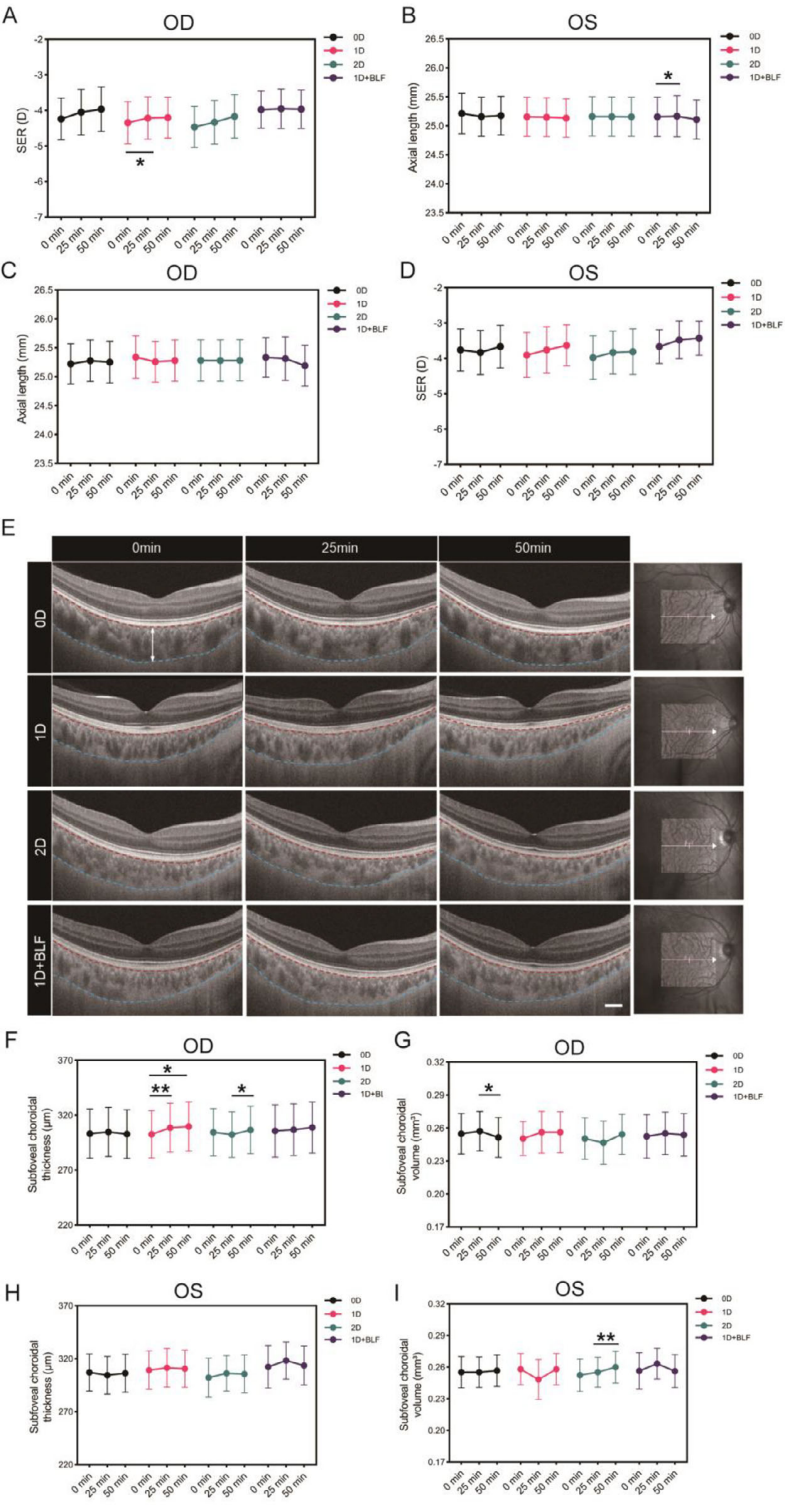
Comparison of AL, SE, and central foveal ChT in different groups during visual tasks. The analysis compares right eye SE and AL (A,C), and left eye AL and SE (B,D) at 0, 25, and 50 min of visual tasks. (E) OCT images from the right eye show central foveal ChT post‐defocusing interventions, marked between red (Bruch's membrane) and blue (sclera) lines, pointed by white arrows. The white line indicates the OCT cross‐section level. (F–I) Statistical comparisons of foveal ChT and volume for both right and left eyes across groups. Student's *t*‐test, mean ± SEM. ^*^ *p* < 0·05, ^**^ *p* < 0·01, ^***^ *p* < 0·001. Scale bar is 200 µm.

Changes in parameters were measured at three time points: before (0 min), during (25 min), and after (50 min) the visual tasks. Figure 2E presents representative OCT images of subfoveal ChT in the right across groups. In the 1D group, ChT increased from 302.52 ± 96.66 to 308.62 ± 99.44 µm at 25 min (Z = −2.94, *P* = 0.003) and to 309.67 ± 100.03 µm at 50 min (Z = −2.87, *P* = 0.004). The 2D group showed a slight increase in ChT after 50 min post‐visual task (Z = −2.23, *P* = 0.026) (Figure 2F). The subfoveal ChT for the left (Figure 2H) showed no significant changes across all groups. The choroidal volume in the right of the 0D group decreased (Z = −1.98, *P* = 0.048) (Figure 2G), while the left in the 2D group showed an increase (t = −3.24, *P* = 0.004) (Figure 2I), indicating differing effects on choroidal volume.

#### 2.1.1 Changes in AL and Spherical Equivalent Refraction (SER)

In the 1D group, the SER for the right eye decreased from −4.35 ± 2.66 to −4.21 ± 2.66 D (t = −2.16, *P* = 0.045) (Figure 2A). In the 1D + BLF group, AL for the left increased from 25.16 ± 1.51 to 25.17 ± 1.54 mm (t = 2.42, *P* = 0.026) (Figure 2B). No significant changes in AL for the right (Figure 2C) or SER for the left (Figure 2D) were observed in the other groups.

### 2.2 Changes in Retinal Defocus

**Figure 3:**
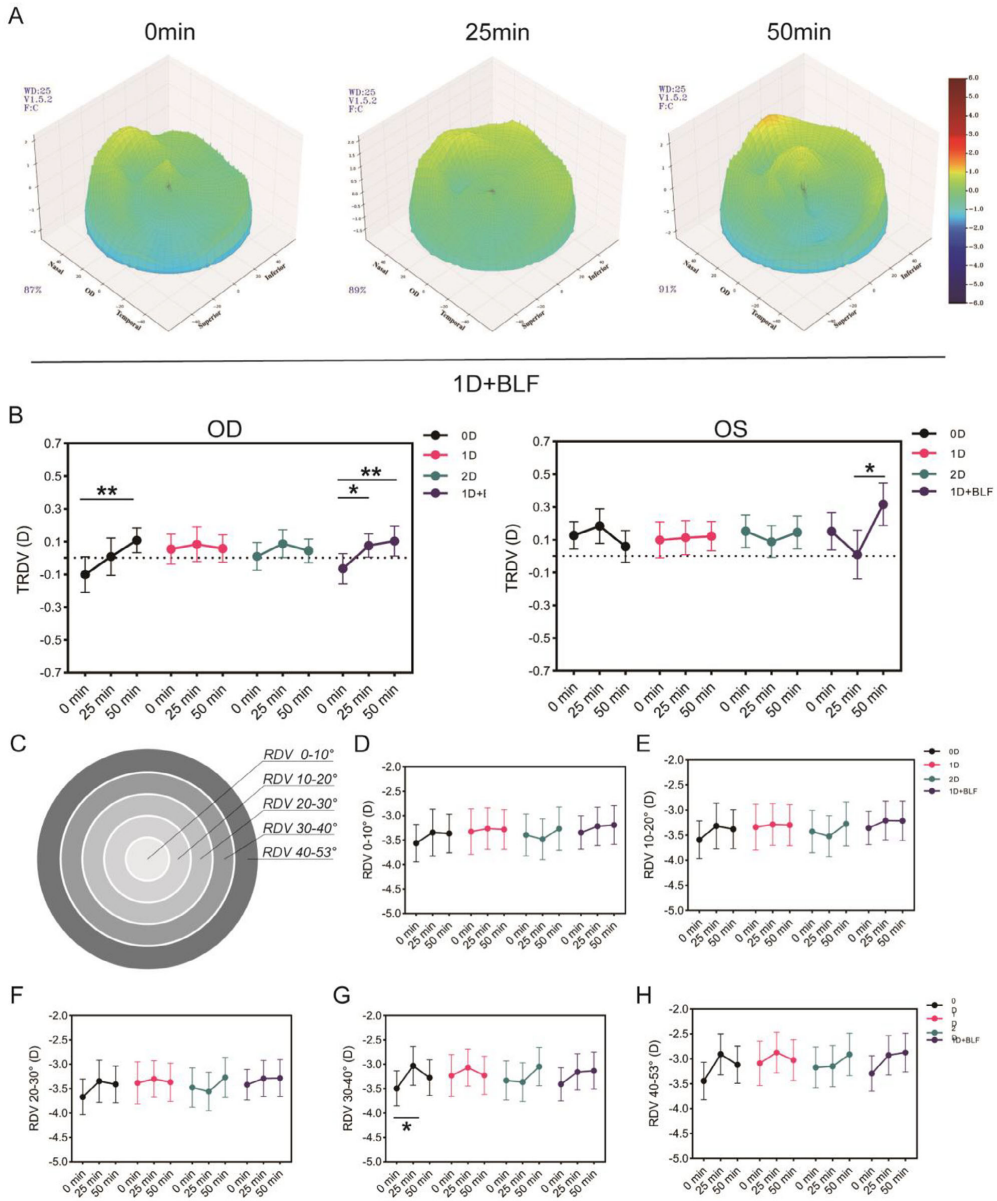
Retinal defocus states in participants across different intervention groups. (A) 3D representation of retinal defocus in the right eye, color‐coded by refractive error intensity, with the 1D+BLF group illustrating the progression over time. Red indicates hyperopic defocus, while blue suggests myopic defocus. (B) Statistical comparison of TRDV at different time points among the groups was presented. (C) Schematic representation of the retinal area division in the fundus, illustrating the mapping of the central and peripheral regions of the retina. (D–H) Comparative analysis of retinal defocus values at various angular sectors during visual tasks for different time points across groups. The central foveal region (0–10°) is depicted in (D), with progressive peripheral sectors shown in (E) (10°–20°), (F) (20°–30°), (G) (30°–40°), and (H) (40°–53°). Student's *t*‐test, mean ± SEM. ^*^ *p* < 0·05, ^**^ *p* < 0·01.

Figure 3A illustrates the real‐time TRDV in the right of the 1D + BLF group at various time points. The 0D group exhibited myopic defocus in the right shifted to hyperopic defocus after the task (TRDV increased from −0.10 ± 0.48 to 0.11 ± 0.33 D, t = −2.98, *p* = 0.007). Both eyes in the 1D + BLF group displayed a trend toward hyperopic defocus, with statistically significant changes in TRDV (OD: t = −2.92, *P* = 0.009; OS: t = −2.75, *P* = 0.012) (Figure 3B). Further analysis of defocus changes in different regions in the right (0°–10°, 10°–20°, 20°–30°, 30°–40°, 40°–53°) indicated no significant changes in the central region (0°–20°) (Figure 3C–E). However, the 30°–40° range of the 0D group exhibited hyperopic defocus, with TRDV changes primarily originating from this area (Figure 3F–H). The 1D group observed a trend toward myopic defocus in the 40°–53° range (t = −2.42, *P* = 0.025). The defocus changes in the same region for the 1D + BLF group were consistent with the TRDV trend (t = −2.14, *P* = 0.044; t = −2.26, *P* = 0.035).

### 2.3 Reading Ability and Visual Fatigue

**Figure 4:**
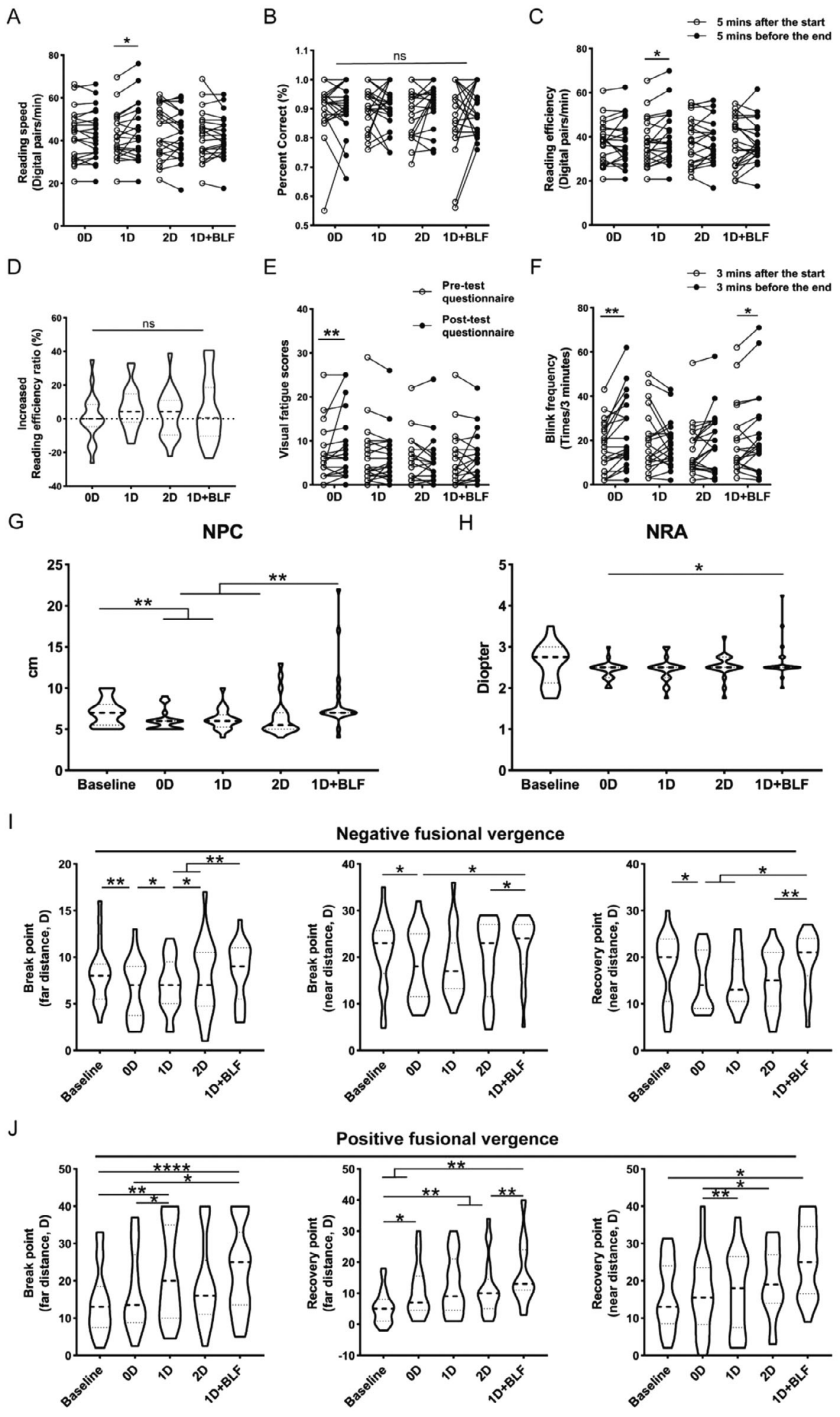
Comparison of text reading performance and visual function among participants in different defocus groups. Comparison of text reading speed (A), reading accuracy (B), and reading efficiency (C) at 5 min into and the last 5 min of the visual task across different groups. (D) Comparison of the change in reading efficiency before and after the visual task among different groups. (E) Changes in visual fatigue scores before and after the visual task for different groups. (F) Comparison of blinking frequency changes during the first and last 3 min of the visual task among groups with different defocus interventions. Comparisons of the NPC (G) and NRA (H) at baseline and following various defocusing conditions, respectively. Comparison of the break and recovery points of negative fusional vergence (I) and positive fusional vergence (J) at both far and near distances for the baseline and different intervention groups. Student's *t*‐test, mean ± SEM. ^*^ *p* < 0·05, ^**^ *p* < 0·01, ^****^ *p* < 0·0001.

In the 1D group, reading speed increased from 41.77 ± 11.01 to 44.30 ± 13.13 digits per minute (t = −2.36, *P* = 0.029). Reading efficiency increased from 37.61 to 39.83 ± 11.62 digits per minute (t = −2.34, *P* = 0.030). No significant differences were observed in the other groups (Figure 4A,C). There was no significant difference in reading accuracy among the groups (Figure 4B). Comparisons of the proportional increase in reading speed across different groups revealed no significant differences (F = 0.534, *P* = 0.660) (Figure 4D).

The 0D group experienced an increase in fatigue scores from 5.0 (2.0, 8.0) to 6.0 (2.0, 11.5) after the task (Figure 4E). Blink frequency significantly increased from 17.29 ± 11.15 to 24.05 ± 15.64 blinks per minute (t = −3.96, *P* = 0.001). Additionally, the 2D group showed a notable increase in blink frequency from 12.0 (10.0, 26.0) to 17.0 (10.0, 30.5) blinks per minute (Z = −2.04, *P* = 0.042) (Figure 4F).

### 2.4 Visual Function Comparison

The near point of convergence (NPC) significantly decreased in the 0D (6.02 ± 1.21 cm), 1D (6.21 ± 1.26 cm), and 2D (6.45 ± 2.28 cm) groups compared to baseline (7.02 ± 1.56 cm) and post‐task in the 1D+1BLF group (8.43 ± 3.93 cm). The 0D group had the lowest near point accommodation (NRA) post‐task (+2.44 ± 0.22 D) (*P* = 0.041). Negative fusional vergence points were significantly lower in the 0D, 1D, and 2D groups compared to baseline and the 1D+BLF group, while positive fusional vergence points increased significantly in all groups (Figure 4G–J). Other visual function indicators showed no statistical differences between groups (Table S1; Table 2).

**Table 2:** The multiple regression analysis revealed the relationship between each independent variable and the dependent variable.

	CVI (OS)	NPC	NFV‐BP (far distance)	PFV‐RP (far distance)	PFV‐RP (near distance)
*Age (years)*					
B	0.025	0.310	−0.015	−0.359	0.366
Beta	0.150	0.315	−0.012	−0.117	0.087
95 % CI	−0.010–0.060	0.110–0.511	−0.241–0.212	−0.999–0.281	−0.583–1.316
*P*	0.156	0.003	0.898	0.267	0.445
*Gender (M/F)*					
B	0.155	1.289	0.839	−3.078	0.458
Beta	0.169	0.239	0.129	−0.184	0.020
95 % CI	−0.035–0.346	0.198–2.381	−0.396–2.074	−6.565–0.409	−4.717–5.632
*P*	0.109	0.021	0.180	0.083	0.861
*Dominant Eye*					
B	0.072	−1.090	−2.518	−0.347	−3.418
Beta	0.074	−0.193	−0.368	−0.020	−0.141
95 % CI	−0.125–0.268	−2.214–0.034	−3.790–1.246	−3.938–3.244	−8.747–1.911
*P*	0.469	0.057	<0.001	0.848	0.205
*Right eye SER (D)*					
B	<−0.001	0.282	1.351	−0.754	0.261
Beta	−0.002	0.271	1.072	−0.233	0.058
95 % CI	−0.099–0.099	−0.285–0.848	0.710–1.992	−2.563–1.056	−2.425–2.946
*P*	0.94	0.325	<0.001	0.409	0.847
*Defocus processing*					
B	0.136	0.732	0.575	1.828	3.032
Beta	0.335	0.307	0.199	0.246	0.296
95 % CI	0.056–0.216	0.273–1.192	0.054–1.095	0.359–3.296	0.852–5.211
*P*	0.001	0.002	0.031	0.015	0.007
R‐square	0.242	0.282	0.372	0.241	0.124
n	21	21	21	21	21

^a^ M: male;

F: female;

SER: spherical equivalent refraction;

NPC: near point of convergence;

OS: left eye;

CVI (OS): choroidal volume increase (OS);

NFV‐BP (far distance): negative fusional vergence break point (far distance);

PFV‐RP (far distance): positive fusional vergence recovery point (far distance);

PFV‐RP (near distance): positive fusional vergence recovery point (near distance);

B: unstandardized coefficients B;

Beta: standardized coefficients beta;

95 % CI: 95 % confidence interval for B;

*P*: *p* value

### 2.5 Linear Regression Analysis on Defocusing Effects

A linear regression analysis assessed the relationship between defocus and dependent variables (changes in ChT and NPC), adjusting for confounding factors such as age, gender, and dominant eye (Table 2). Regression analysis revealed significant associations between defocus and the changes in the left choroidal volume, NPC, negative fusion break points, and positive fusion recovery points. The explanatory power of defocus for these parameter changes was 24.2 % (R^2^ = 0.242, *P* = 0.001), 28.2 % (R^2^ = 0.282, *P* = 0.002), 37·2 % (R^2^ = 0.372, *P* = 0.031), 24·1 % (R^2^ = 0.241, *P* = 0.015), and 12.4 % (R^2^ = 0.124, *P* = 0.007), respectively. Other independent variables (age, sex, dominant eye) showed no significant correlation with the outcome measures across the groups.

## 3 Discussion

This study investigated the effects of varying degrees and intensities of blue light defocus on ocular physiological parameters in adults following visual tasks. Significant alterations were observed in refractive power, AL, ChT, and retinal defocus state, particularly notable in the 1D and 1D + BLF groups. These findings suggest that short‐term near‐distance use of small‐screen devices may be associated with increased myopia risk, whereas blue light defocus might help mitigate this risk. Furthermore, the 1D defocus group showed improved reading speed, while the 0D group exhibited marked visual fatigue, highlighting the potential benefits of defocus. These results provide critical insights into the roles of blue light defocus in refractive states and ocular health.

The SER results showed that participants in the 1D defocus group experienced a transient reduction in refractive error. Previous research [10, 17, 20] has established that medium to long wavelengths of light promote axial elongation, whereas blue light leads to reduced AL. These findings have been corroborated through rigorous experiments on humans, chicks, and guinea pigs [21]. Consistent with prior findings, our study showed that blue light‐induced myopic defocus effectively reduced axial elongation. Notably, the blue light partially filtered group showed a statistically significant increase in AL, suggesting that reducing blue light intensity diminished its effect. Although no significant changes were observed in the other groups, this could be attributed to the precision of the measuring equipment and lighting conditions. Overall, these findings underscore the impact of blue light on axial growth, providing new insights for the prevention and treatment of myopia.

ChT is a critical biological indicator in regulating AL [7]. A reduction in ChT and blood flow can lead to scleral hypoxia, promoting axial elongation [22]. Conversely, increased ChT suppresses axial growth and protects against myopia [23, 24]. Our study found that blue light defocus significantly increased ChT in the 1D and 2D groups, indicating improved ocular blood flow and potentially reduced hypoxia, which may help prevent visual damage from prolonged screen use and lower myopia risk. Prior research suggested that blue light positively influences ocular growth by stimulating dopamine release in the retina [25, 26].

Retinal peripheral defocus significantly influences axial changes [27], with periphery hyperopic defocus typically increasing as myopia progression [28]. Our experiment revealed that this trend was particularly pronounced in the 0D group, where both eyes tended toward hyperopic defocus. In contrast, the 1D and 2D groups did not show a relative increase in hyperopic defocus. Furthermore, the study revealed that peripheral myopic defocus was required to exceed a certain threshold to effectively mitigate myopia progression. When analyzing defocus statuses across different regions, no significant changes were observed in the 0°–20° range for the 0D group, while the 20°–53° range leaned toward hyperopic defocus. Specifically, RDV 40°–53° in the 1D group showed a tendency toward myopic defocus, while the 1D+BLF group was inclined toward hyperopic defocus in the same range, reflecting the influence of blue light filtering on defocus effects.

The 1D defocus group exhibited a marked improvement in reading speed and efficiency, consistent with previous studies [29, 30] hypothesizing that moderate defocus might enhance visual perception. While no significant differences in task accuracy were observed among the groups, participants in the 0D group experienced increased visual fatigue, with a notable rise in blink frequency in the 0D and 2D groups. This validated that appropriate defocus alleviated visual fatigue induced by visual tasks. The defocus effect also influences the eye's accommodation and convergence abilities [31]. Regression analysis demonstrated a substantial relationship between defocus and improvements in visual function, consistent with established findings on ocular accommodation and convergence. Previous research indicated that prolonged near work can induce accommodative spasm, affecting the NPC [32, 33]. Post‐defocus, participants exhibited decreased accommodation demands and increased convergence reserves.

Our primary objective was to characterize the immediate effects of blue‐channel defocus on retinal hemodynamics, AL, and SER during short‐term intensive near work. In a context where transient AL elongation and a myopic shift are typically observed, the attenuation or reversal of these trends in AL and SER, accompanied by ChT thickening, aligns with established physiological mechanisms. Although the short‐term effects are modest in magnitude, their coherent direction under intensive near work, together with ChT thickening and measurable improvements in visual performance (12 % faster reading and 40 % less visual fatigue in the 1D group), supports physiological relevance and indicates potential patient‐relevant benefits.

Although this study evaluated the effects of blue light defocus technology on ocular health and refractive status in adults, its potential implications for pediatric myopia management warrant cautious interpretation. We observed a reduction in SER from −4.35 D to −4.21 D (p = 0.045), along with choroidal thickening in both the 1D and 2D defocus groups, consistent with existing evidence that peripheral defocus signals can modulate axial elongation—also a key mechanism in childhood myopia progression. Compared with adults, children exhibit greater scleral extensibility and more pronounced axial changes. Therefore, for pediatric and adolescent populations, it is necessary to systematically assess the magnitude of blue light defocus introduced by screen viewing and to determine whether, and to what extent, short‑wavelength blue‑light filtering should be implemented. These insights should guide the optimization of spectral modulation parameters to achieve an appropriate balance between axial control and photoprotection.

There are certain limitations to this study. First, to investigate the impact on early myopic patients, the sample should include younger children. Second, long‐term follow‐up of participants is necessary to assess the sustained effects and long‐term implications of the interventions. Additionally, our research did not encompass assessments across different wavelengths of the light spectrum. Future studies should include larger sample sizes and a broader age range, and perform long‐term tracking and evaluate the effects of different wavelengths on parameters such as AL to provide a more comprehensive perspective and deeper insights.

## 4 Conclusions

In summary, this study established a model of blue light defocus with varying degrees and intensities specific to small screen displays. Through a prospective clinical intervention trial, we explored its effects on critical ocular physiological parameters, including refractive power, AL, retinal defocus, and visual function. Under these experimental conditions, the blue light defocus group attenuated the hyperopic defocus induced by near‐distance work and was associated with increased ChT and potential ocular health benefits, which may contribute to reduced myopia risk. Low‐degree blue light defocus enhanced reading efficiency and alleviated visual fatigue to some extent. Based on the results related to visual function, participants in the various defocus groups exhibited reduced reliance on their own defocusing abilities while increasing their convergence reserves. The diminished effects of post‐blue light filtering further underscore this significance. This study has provided a comprehensive assessment of the influence of different degrees of blue light defocus on ocular refractive power, AL, retinal defocus state, and ChT, potentially laying the groundwork for the development of blue light defocus‐based display devices that aim at promoting ocular health and myopia prevention strategies.

## 5 Experimental Section

### 5.1 Study Design

This study, comprising a randomized crossover trial and a prospective self‐controlled design, was conducted as a single‐blind trial with 21 participants per group, totaling 84 participant‐visits. The study was approved by the Ethics Committee of Beijing Tongren Hospital (Approval No.: TREC2024‐KY067) and is registered with ClinicalTrials.gov (ID: NCT06449976).

### 5.2 Sample Size Calculation

To determine the required sample size, a preliminary pilot study involving all four intervention groups (0D, 1D, 2D, and 1D+BLF) was conducted, with subfoveal choroidal thickness (SFCT) selected as the primary outcome measure. Preliminary test results indicated standard deviations of SFCT differences before and after visual tasks for different groups were 1.47, 8.74, 4.27, and 5.99 µm, with mean differences of 4.2, 6.4, −3.6, and 4.4 µm. A two‐sided significance level(α)of 0.05 and a power of 90 % (β = 90 %) were set, using *Z* as the corresponding statistical measure. The dropout rate was 5 %.

Sample size calculation utilized the formula:

$$n=\frac{{\left(Z_{\alpha /2}+Z_{\beta }\right)}^{2}*\sigma^{2}}{\delta^{2}}$$

Based on the overall standard deviation (σ) and mean difference (δ), n ≥20 participants were required. Finally, a total of 24 participants were enrolled in the study, and 21 participants finished the study.

### 5.3 Participants

This study involved 21 adults aged 20–30 years. Inclusion criteria required participants to have no systemic diseases or ocular conditions, with astigmatism ≤2.0D and anisometropia <1.5D. Best‐corrected visual acuity (BCVA) was ≤ 0.0 logMAR (Table 1). Participants had no history of other myopia interventions and wore only single‐vision spectacles. To minimize caffeine effects, participants were instructed to avoid caffeinated beverages on the test day [34, 35].

### 5.4 Randomization and Masking

Participants were randomly assigned to four groups of visual task tests, 0D, 1D, and 2D groups, to investigate the effects of varying blue channel defocusing, with 30 % blue light filtration in the 1D (1D+BLF) group to confirm its influence, totaling 84 person‐times. SPSS software was used to generate random integer values between 0 and 3, with 0 corresponding to the control group (no defocus treatment), 1 to the 1D group, 2 to the 2D group, and 3 to the 1D+BLF group. Blinding was implemented for all participants, and if a participant drew a previously assigned number, the selection was repeated until a new, unique number was chosen.

### 5.5 Experimental Equipment and Environment

**Figure 5:**
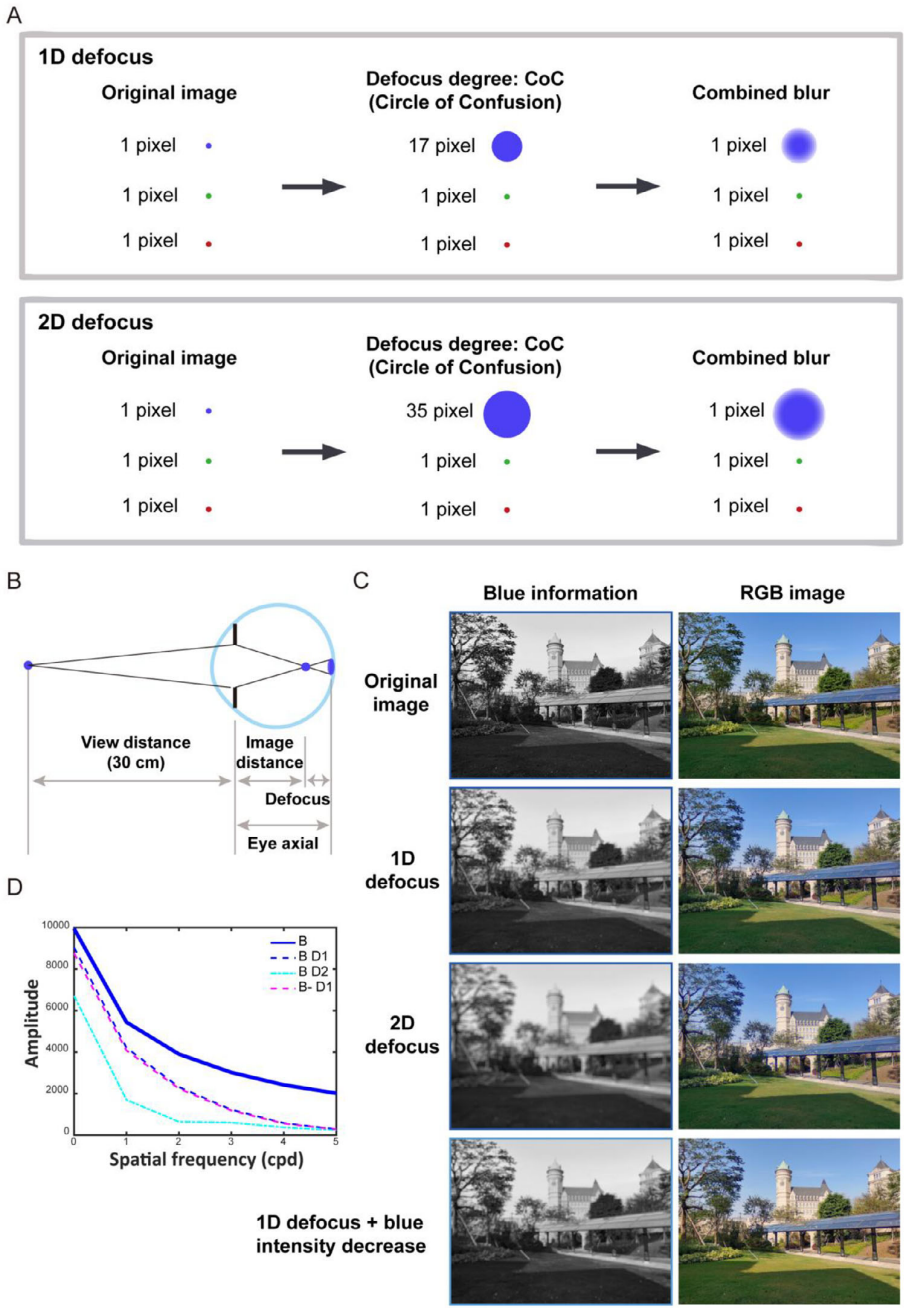
Schematic illustration of different interventions of the blue channel and its effects on visual perception. (A) Illustration of varying degrees of defocus intervention applied to the blue channel, demonstrating the progression of the CoC and combined blur of pixels. (B) Schematic representation of ocular defocus when participants view a display device from a distance of 30 cm. (C) Presentation of images after different degrees of blue channel processing. (D) The summed signal amplitude of image blue information as a function of spatial frequency and defocus, indicating how the intensity of the blue channel is attenuated with increasing defocus and spatial frequency.

Visual tasks were administered using a 6.7 inch smartphone (2664 × 1200 pixels^2^) positioned on a stable desktop at 0.75 meters height. Defocus was achieved through adjustments in the Circle of Confusion (CoC) to quantify the blur. Based on the principle of the CoC, when light rays fail to converge to a single point, they form a diffused circular projection on the imaging plane, known as the circle of confusion. By adjusting the size of the circle of confusion for blue light in the display device, we can simulate the defocus effects of blue light under different visual tasks. For instance, the CoC for blue light was enlarged to 17 pixels with 1D defocus and to 35 pixels with 2D defocus. The study established four groups: 0D, 1D, and 2D groups to investigate the effects of varying blue channel defocusing, with 30 % blue light filtration in the 1D (1D+BLF) group to confirm its influence. All tests were conducted in a darkroom environment with light intensity limited to 5 lx. Figure 5 illustrates the effects of varying degrees of blue channel defocusing on visual clarity and signal strength.

Refractive errors were assessed non‐cycloplegically using an auto‐refractor (Nidek ARK‐510A, Japan). AL was measured with IOLMaster‐700 (IOLMaster 700, Carl Zeiss Meditec AG, Germany). ChT and choroidal volume were evaluated using optical coherence tomography (OCT; YG‐100K PRO, TowardPi Medical Technology Ltd, China). Retinal defocus values (RDV and TRDV) were obtained with a multispectral fundus camera (MSI C2008, Shenzhen Thondar Technology Co., Ltd, China). Visual function parameters were assessed with a comprehensive refractometer, and blink frequency changes were recorded using high‐definition video.

### 5.6 Experimental Procedure

An overview of the participants’ flow through the study is given in Figure 1. Each participant was randomly assigned to a different group each day over four consecutive days, ensuring all completed experiments in all groups (Figure S1). A 5 min “rest period” preceded each test to minimize the effects of prior visual activities. Baseline visual function, including accommodative and convergence functions, was assessed.

The visual‐task protocol is illustrated in the accompanying figure. The entire session lasts 50 min: 0–5 min and 45–50 min are devoted to reading text, consisting of randomly paired Roman numerals presented in a uniform font size. From 5–45 min, participants watched an animated feature with a typical, broad color and luminance distribution, ensuring the stimulus reflected normal viewing content while maintaining consistency across participants. The sequence, testing intervals, and viewing distance are standardized across all participants.

During the first and last 5 min of the visual task, participants read texts with varying defocus to evaluate reading efficiency. This involved identifying pairs of Roman fonts (12 points) and determining whether the presented numerals matched reference digits. Reading speed was calculated based on correctly identified numeral pairs per minute, while accuracy was the ratio of correctly identified pairs to the total within a 5 min interval. Reading efficiency was defined as the product of reading speed and detection rate.

The middle 40 min involved watching videos with consistent content. Assessments were conducted at the 0 min (beginning), 25 min (midpoint), and 50 min (end) marks of the visual task. For each participant, refractive error, AL, ChT, and retinal defocus were assessed at all three times. Visual function assessments were conducted before and after each visual task intervention in every group. The visual function parameters measured in this study mainly included accommodative and vergence functions. Specifically, these included the accommodative convergence/accommodation (AC/A) ratio, horizontal heterophoria, fusional vergence, accommodative sensitivity, binocular cross‐cylinder (BCC), and positive relative accommodation (PRA). The right eye was always measured first, followed by the left eye, to ensure consistency. Participants completed the measurements within 5 min. All examinations were conducted individually by an experienced operator.

### 5.7 Statistical Analysis

Data were analyzed using IBM SPSS Statistics for Windows, version 26.0 (IBMCorp., Armonk, N.Y., USA). Normality tests were performed, with normally distributed data described as mean ± standard deviation and non‐normally distributed data as median (Q1, Q3). Paired *t*‐tests evaluated reading efficiency and visual fatigue before and after the tasks. Repeated measures, ANOVA, and non‐parametric tests were used for other data comparisons. The significance level was adjusted using the Bonferroni method to account for multiple testing. Linear regression analysis explored the association between defocus intervention and dependent variables (changes in ChT and NPC) with the adjustment of confounding factors, including age, gender, and dominant eye. A *p*‐value of less than 0.05 was considered statistically significant.

### 5.8 Statement

All participants consented to take part in this research work.

## Funding

This work is partly supported by the National Natural Science Foundation of China (82125007), The Youth Beijing Scholar program, and the Beijing Municipal Public Welfare Development and Reform Pilot Project for Medical Research Institutes (PWD&RPP‐MRI, JYY2023‐6).

## Conflicts of Interest

The authors declare no conflicts of interest.

## Supporting information




**Supporting file**: gch270064‐sup‐0001‐SuppMat.docx

## Data Availability

The datasets collected during this study are available from the corresponding author upon reasonable request. For further details or inquiries regarding the data, please contact the corresponding author.
