# Supplementary material for: Blue Light Defocus Induces A Positive Effect on Refractive Status and Ocular Health: A Randomized Crossover Trial
Source: Glob Chall. 2025 Nov 14;9(12):e00222. doi: 10.1002/gch2.202500222 (PMC12697078; doi:10.1002/gch2.202500222)
Supplement: Supplementary file 1 — Supporting file: gch270064‐sup‐0001‐SuppMat.docx [file GCH2-9-e00222-s001.docx]

**Supplement Figure**


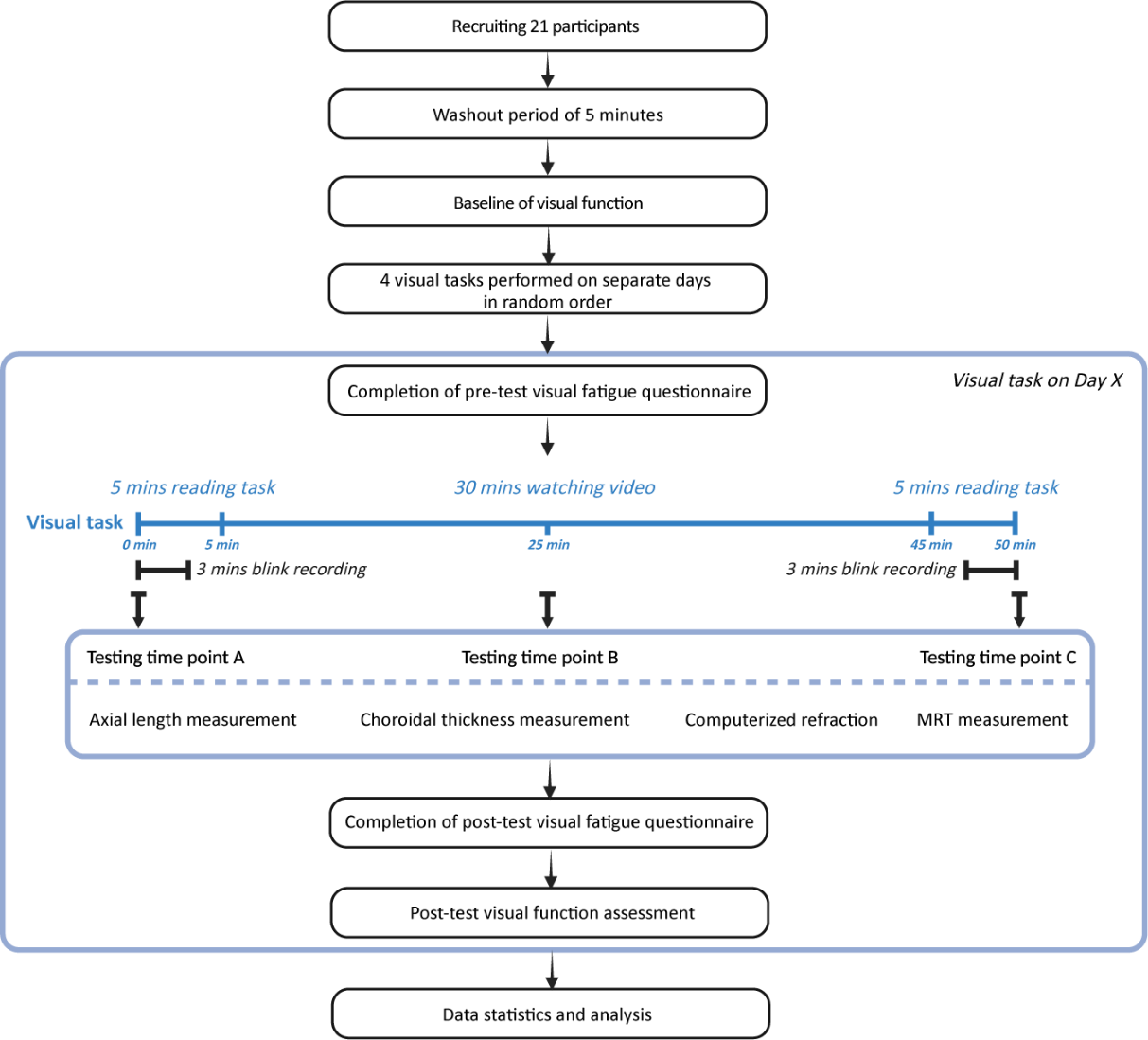


**Supplement Figure.** Illustrates the detailed procedure, including the composition of the 50-minute visual task and the arrangement of various testing time points.

**Supplement Tables**

**Supplement table 1. Comparison of AC/A, horizontal heterophoria, and fusional vergence among different defocus treatment groups.**

| **Time** | **Calculated AC/A** | **Horizontal heterophoria (near distance), △** | **Horizontal heterophoria (far distance), △** | **Positive fusional vergence (BO), D** |  | **Negative fusional vergence (BI), D** |
| --- | --- | --- | --- | --- | --- | --- |
|  |  |  |  | **Break point (near distance)** |  | **Recovery point (far distance)** |
| Before test | 2·49±1·42 | -5·33±8·16 | -0·74±3·76 | 27·37±10·17 |  | 4·95±2·61 |
| 0D | 2·29±1·90 | -7·50(-9·75, -2·50) | -1·19±3·94 | 22·74±10·59 |  | 4·33±2·80 |
| 1D | 1·09±2·10 | -6·94±5·14 | -0·91±4·12 | 28·00(10·75, 37·50) |  | 4·45±2·79 |
| 2D | 1·94±2·59 | -5·56±6·46 | -0·74±3·62 | 27·67±10·25 |  | 4·74±3·07 |
| 1D+BLF | 3·11±1·37 | -6·00(-9·25, -2·50) | 0·38±3·88 | 30·00(23·00, 38·00) |  | 6·00±2·89 |
| *F/H* | 1·256 | 0·376 | 0·395 | 4·850 |  | 1·095 |
| *P* | 0·293 | 0·984 | 0·811 | 0·303 |  | 0·363 |

*n*=21 cases. χ2 and F: The statistical value of Friedman test and repeated measurement analysis of variance respectively. *P*, significance values from one-way ANOVA.

**Supplement table 2. Comparison of accommodative sensitivity, BCC, and PRA across different defocus treatment groups.**

| **Time** | **Flipper (OD), cpm** | **Flipper (OS), cpm** | **Flipper (OU),** **cpm** | **BCC, D** | **PRA, D** |
| --- | --- | --- | --- | --- | --- |
| Before test | 10·50±3·16 | 10·60±2·93 | 10·36±3·54 | 0(0·00, 0·25) | -3·55±1·32 |
| 0D | 10·48±3·38 | 10·26±3·30 | 9·60±2·92 | 0(0·00, 0·38) | -3·75(-4·63, -2·38) |
| 1D | 13·25±3·11 | 14·81±2·44 | 10·60±3·50 | 0(0·00, 0·50) | -3·20±1·84 |
| 2D | 12·44±3·57 | 13·69±2·87 | 10·50±3·17 | 0(0·00, 0·25) | -3·50(-4·25, -3·00) |
| 1D+BLF | 10·55±2·08 | 10(9·25, 13·00) | 10·10±2·00 | 0·19±0·47 | -3·32±1·14 |
| *F/H* | 0·133 | 2·219 | 0·339 | 1·227 | 0·367 |
| *P* | 0·970 | 0·696 | 0·851 | 0·874 | 0·985 |

*n*=21 cases. χ2 and F: The statistical value of Friedman test and repeated measurement analysis of variance respectively. *P*, significance values from one-way ANOVA. BCC: binocular cross cylinder; PRA: positive relative accommodation; OD: right eye; OS: left eye; OU: both eyes; cpm: cycles per minute.
